# ‘Omics’ and Plant Responses to *Botrytis cinerea*

**DOI:** 10.3389/fpls.2016.01658

**Published:** 2016-11-15

**Authors:** Synan F. AbuQamar, Khaled Moustafa, Lam-Son P. Tran

**Affiliations:** ^1^Department of Biology, United Arab Emirates UniversityAl Ain, UAE; ^2^Conservatoire National des Arts et MétiersParis, France; ^3^Plant Abiotic Stress Research Group & Faculty of Applied Sciences, Ton Duc Thang UniversityHo Chi Minh City, Vietnam; ^4^Signaling Pathway Research Unit, RIKEN Center for Sustainable Resource ScienceYokohama, Japan

**Keywords:** *Arabidopsis*, biotic stress, *Botrytis cinerea*, omics, metabolomics, proteomics, transcriptomics

## Abstract

*Botrytis cinerea* is a dangerous plant pathogenic fungus with wide host ranges. This aggressive pathogen uses multiple weapons to invade and cause serious damages on its host plants. The continuing efforts of how to solve the “puzzle” of the multigenic nature of *B. cinerea*’s pathogenesis and plant defense mechanisms against the disease caused by this mold, the integration of omic approaches, including genomics, transcriptomics, proteomics and metabolomics, along with functional analysis could be a potential solution. Omic studies will provide a foundation for development of genetic manipulation and breeding programs that will eventually lead to crop improvement and protection. In this mini-review, we will highlight the current progresses in research in plant stress responses to *B. cinerea* using high-throughput omic technologies. We also discuss the opportunities that omic technologies can provide to research on *B. cinerea*-plant interactions as an example showing the impacts of omics on agricultural research.

## Introduction

*Botrytis cinerea*, often known as gray mold, is a necrotrophic fungal pathogen that kills its host plant cells, and then colonizes the dead tissues. It can infect more than 200 crop hosts, causing enormous economic damage on important crops, such as tomatoes, berries and petunia flowers (Fernández-Acero et al., 2011; Elad et al., 2015). As a result, *B. cinerea* has been considered the second most dangerous phytopathogen worldwide (Dean et al., 2012). The long-established approach of treating infections caused by this pathogen has been the use of large amounts of fungicides during the seasonal crop cycle (De Miccolis Angelini et al., 2014). This method, however, has become impractical due to the development of fungal resistance and the public health safety concerns associated with fungicide uses. Recently, huge efforts have been made to engineer resistant crop plants to *B. cinerea* in an environmentally sustainable, safe and cost-effective manner.

As an opportunist fungus, *B*. *cinerea* attacks weak, damaged or senescent tissues through wound or previously infected sites (Elad and Evensen, 1995). *B. cinerea* has developed sophisticated penetration, infection and colonization strategies to suppress plant defenses (for review, see van Kan, 2006), which are mediated by lytic enzymes, toxins, stress-induced reactive oxygen species (ROS), necrosis-secreted proteins and a large group of secondary metabolites (Choquer et al., 2007). On the other hand, plant defense mechanisms can restrain these strategies through preformed (constitutive) or induced (physical and chemical) barriers (for review, see Mengiste et al., 2010). Plant cuticle and cell wall serve as the first line of defense against this pathogen (Chassot et al., 2007; AbuQamar et al., 2013; AbuQamar, 2014). Chemical defenses, such as the constitutively present phytoanticipins and phytoalexins that are produced *de novo* upon infection, also provide protection (VanEtten et al., 1994). Pathogenesis-related (PR) proteins, defensins, antimicrobial compounds are accumulated in response to infection (van Loon and Van Strien, 1999; van Loon et al., 2006). Moreover, phytohormones, including salicylic acid, jasmonic acid, ethylene, abscisic acid (ABA), brassinosteroids, auxin, cytokinins, gibberellins and strigolactone, contribute, individually or co-operately, in mediating plant responses to *B. cinerea* (Thomma et al., 1998; Audenaert et al., 2002; Ferrari et al., 2003; Torres-Vera et al., 2014; AbuQamar et al., 2016).

**FIGURE 1:**
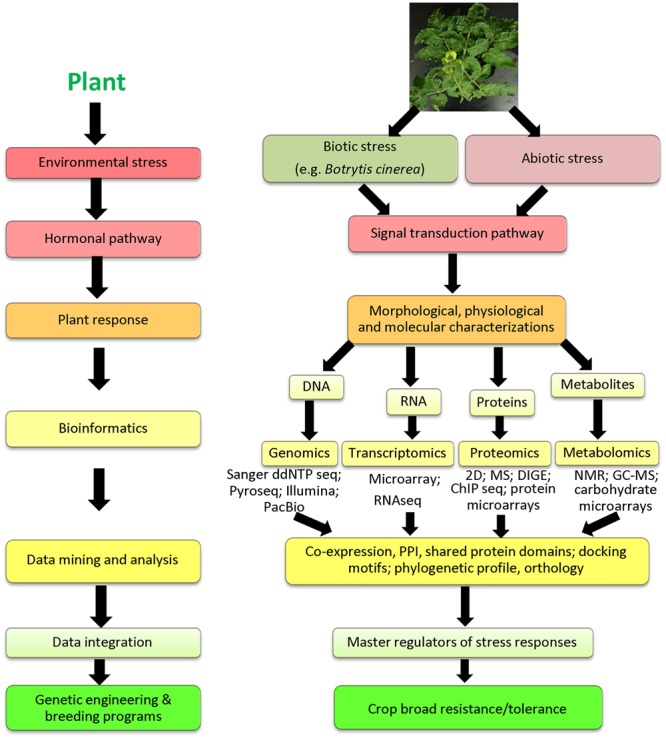
**Schematic representation of omic approaches used in improvement of plant resistance/tolerance to biotic and abiotic stresses.** Sanger ddNTP seq, Sanger dideoxy nucleotide; Pyroseq, pyrosequencing; Illumina, Illumina sequencing; PacBio, Pacific Biosciences; RNAseq, RNA sequencing; ChIP seq, chromatin immunoprecipitation sequencing; 2D, 2-dimensional gel electrophoresis; DIGE, differential gel electrophoresis; MS, mass spectrometry; NMR, nuclear magnetic resonance; GC-MS, gas chromatography-mass spectrometry; PPI, protein-protein interaction.

Recent research technologies have developed efficient omic tools to unravel the molecular mechanisms of plant responses to *B. cinerea* and to improve disease diagnosis and fungal detection. Genome is a complete set of chromosomes, which contains all genes in an organism. Transcriptome describes the entire set of coding and non-coding RNAs, whereas proteome is the collection of proteins derived from a genome. Metabolome are all metabolites found in a biological system (e.g., cell, tissue, organ, or organism). Advances in high-throughput DNA sequencing, RNA sequencing (RNAseq), mass spectrometry (MS), and nuclear magnetic resonance (NMR) at the genomic, transcriptomic, proteomic and metabolomic levels, and through the multi-omics (also known as integrated-omics) (Shiratake and Suzuki, 2016), have made possible the development of such data into a systems biology-based framework (**Figure 1**). High-throughput-next generation sequencing (HT-NGS) technologies, ranging from RNAseq to whole-genome sequencing, are fast, sensitive and accurate tools for detection of *B. cinerea* genome from symptomatic or asymptomatic plants and understanding defense mechanisms associated with fungal infections *in planta* (Smith et al., 2014). Moreover, HT-NGS techniques have promising applications at the molecular plant-*B. cinerea* interaction research. Applications of advanced technologies have enabled us to gain insights into fungal genome variability, pathogenic diversity, host range and evolution within *B. cinerea*’s host plant. Availability of omic data will substantially advance our understanding of *B. cinerea* infection strategies, thereby enhancing future predictions of plant responses to the gray mold disease (Hahn et al., 2014; Jiang et al., 2016). In this review, we will overview the recent applications and impacts of omic technologies on agricultural research, focusing on plant-*B. cinerea* interactions. Future studies should be directed at moving from smaller (laboratory) scales using omic tools to larger (field) scales using genetic engineering and breeding strategies to develop low cost and durable disease-resistant crops.

## Genomics

DNA sequencing approaches remain cost-effective, and both the traditional Sanger dideoxy nucleotide sequencing and pyrosequencing have proved their success for *de novo* and confirmatory sequencing (Pareek et al., 2011). Pyrosequencing is usually used for single nucleotide polymorphism (SNP) analysis and sequencing of short stretches of DNA (Fakruddin and Chowdhury, 2012). The NGS technologies -Illumina/Solexa, Ion Torrent Personal Genome Machine (PGM) and Pacific Biosciences (PacBio) sequencing methods- have revolutionized genomic and genetic research (for review, see Heather and Chain, 2016). *B. cinerea* has become a model for dissecting the complexity of necrotrophs and broad host-range pathogenicity. *B. cinerea* strains may survive different environmental stresses that inhibit or promote infections on their host plant (Ahlem et al., 2012). To gain an in-depth understanding of *B. cinerea*-plant interactions at whole genome level, Syngenta AG initiated genome sequence project for *B. cinerea* T4 and B05.10 strains, obtaining a genome size of 37.9 Mbp (14,270 genes) and 38.8 Mbp (13,664 genes), respectively, using Illumina HiSeq2000 technology (Amselem et al., 2011; Staats and van Kan, 2012). A recent report using a combination of two sequencing technologies, namely Illumina and PacBio, has assembled a gapless genome sequence of *B. cinerea* strain B05.10 (van Kan et al., 2016). This assembly is comprised of 18 chromosomes, a genetic map of 4153 centimorgan and approximately 75,000 SNP markers. Comparative analysis of the genome sequences revealed high sequence identity and gene arrangement similarity between *B. cinerea* and *Sclerotinia sclerotiorum*, but different mating behavior and compatibility systems between these pathogenic fungi (Amselem et al., 2011), suggesting no unique features that distinguished them as pathogenic and saprotrophic fungi. Differences in the number and diversity of secondary metabolism (SM) gene clusters are key distinctions between the genomes of these two pathogenic species, and thus attributing to their differential environmental habitats. For example, *S. sclerotiorum* produces 28 SM enzymes, whereas *B. cinerea* secrets 43. In addition, botrydial, botcinic acid, and ABA biosynthetic genes were characterized in *B. cinerea*, but not in *S. sclerotiorum*. The regulation of sexual reproduction, content of transposable elements, and the sequence and organization of mating-type (MAT) loci between the two species have also been distinguished. Yet the genomes of the two species show high sequence identity and similar gene arrangements, implicating no unique features between the genomes of *S. sclerotiorum* and *B. cinerea*, which could be distinguished as a “clear-cut” evidence of their aggressive behavior and multigenic nature of pathogenesis.

A draft genome of *B. cinerea* BcDW1 strain, isolated from botrytized grapes, was also sequenced (Blanco-Ulate et al., 2013). Candidate secreted proteins involved in plant tissue penetration and decomposition, including glycoside hydrolases, carbohydrate esterases, and polysaccharide lyases were identified as compared with T4 and B05.10 genomes. Other secreted laccases and carboxylesterases were also detected for their relevance to noble rot (Magyar, 2011). Comparative analyses of the genome sequences of the *B. cinerea* strains have been proved to be useful for elucidating the genetic and environmental bases of *B. cinerea*-host specificity (Atwell et al., 2015). The genomes of 13 different *B. cinerea* isolates have been re-sequenced to measure their genetic diversities, which pointed out the fact of the broad host range of the species, and their potential ability to adapt to new hosts.

*Arabidopsis* is a small flowering plant that offers important advantages for basic research in genetics and molecular biology. *Arabidopsis* genome sequence was first completed in 2000, with a genome size of ∼135 Mbp (Arabidopsis Genome Initiative, 2000). A full public database of the *Arabidopsis* complete genome, genome maps, genetic and physical markers, gene structure and gene expression, DNA and seed stocks, can be accessed via The Arabidopsis Information Resource (TAIR^1^) database. The “flexibility” of the *Arabidopsis* genome allows this plant to adapt to various environmental conditions. This is evidenced when the 1001 Genomes Project investigated the whole-genome sequence variation among 100 *Arabidopsis* ecotypes from different geographical regions (Cao et al., 2011). The first full genome sequence of tomato (*Solanum lycopersicum*) “Heinz 1706” was achieved in 2012 (Tomato Genome Consortium, 2012). Sequences and arrangements of 35,000 genes on 12 chromosomes have been described. In 2014, the genomes of 360 tomato varieties were also sequenced (Lin et al., 2014). Bolger et al. (2014) have sequenced the stress-tolerant tomato wild species, *S. pennellii*, and identified candidate genes and transposable elements that would play a crucial role in survival in arid habitats. In 2011, the woodland strawberry (*Fragaria vesca*) was sequenced (Shulaev et al., 2011). The relatively small-sized genome (240 Mbp; 35,000 genes) of this perennial plant shares substantial sequence identity with those of the cultivated strawberry (*F.* × *ananassa*) and other rosaceous plants. The genetic map of the grape (*Vitis vinifera*) was completed in 2007 by the shotgun sequencing approach (Jaillon et al., 2007). In addition, genome sequence of chickpea (*Cicer arietinum*) was also assembled by two independent groups (Jain et al., 2013; Varshney et al., 2013; Parween et al., 2015; Thudi et al., 2016). The releases of genome sequences of *B. cinerea* and its hosts have helped identify candidate genes associated with virulence of *B. cinerea* and potential target genes associated with resistance in host crops. The genome sequences of *B. cinerea* hosts have provided us a means to study and gain more insights into plant defense system against this “nasty” fungus.

Genome sequencing will help breeders look at the “blueprint” of crop plants to produce resistant hybrids. As long as the genomes of the fungus and its host plants have been sequenced, a whole-genome gene expression analysis will identify the critical factors in *B. cinerea* pathogenesis and disease resistance mechanisms in plants, pathogen-derived effectors, and the molecular events associated with infection processes *in planta*.

## Transcriptomics

Comparative gene expression analyses can be used to mine the regulatory information through transcriptomic methods to generate data on stress modulations of gene expression in plants. High-throughput methods used for transcriptomics include hybridization-based (microarray technology) and sequencing-based approaches (RNAseq), which allow us to carry out transcriptomic analyses in both model and non-model organisms (Warren et al., 2007). Generally, transcriptomic measures are best suited for early identification of cell responses to an individual or multiple stress(es). Plant responses to *B. cinerea* undergo transcriptional reprograming, showing that over 12% of the *Arabidopsis* genome are differentially expressed genes (DEGs), of which 1498 (7%) and 1138 (5%) were reported to be *B. cinerea*-induced and -repressed genes, respectively (Sham et al., 2014, 2015). A number of DEGs, which were shown to be implicated in *B. cinerea* defense, encode transcription factors, including WRKYs (Birkenbihl et al., 2012; Liu et al., 2015), APETALA2/ethylene response factors (AP2/ERFs) (Son et al., 2012; Maruyama et al., 2013), TGAs (Windram et al., 2012; Sham et al., 2014), NACs (Wang et al., 2009), and MYBs (Ramírez et al., 2011). Several studies focusing on the transcriptional regulation of responses to multiple stresses have identified commonly regulated genes responsive to both *B. cinerea* infection and simultaneous abiotic stresses, such as drought, heat, or salinity, in *Arabidopsis* using microarray analyses (Atkinson and Urwin, 2012; Sham et al., 2015). In tomato, a transcriptomic study using RNAseq has distinguished the natural variation among wild *Solanum* species (Smith et al., 2014). Following *B. cinerea* infection, photosynthetic and metabolic processes were suppressed, whereas defense-related genes, such as those encoding PR protein 1 (PR1), β-1,3-glucanase and subtilisin-like protease, were simultaneously induced in the highly *B. cinerea*-resistant species, *S. lycopersicoides*. Expression of a number of secondary metabolites- and defense-related genes in *S. lycopersicum* were also up-regulated by *B. cinerea* infection.

**FIGURE 2:**
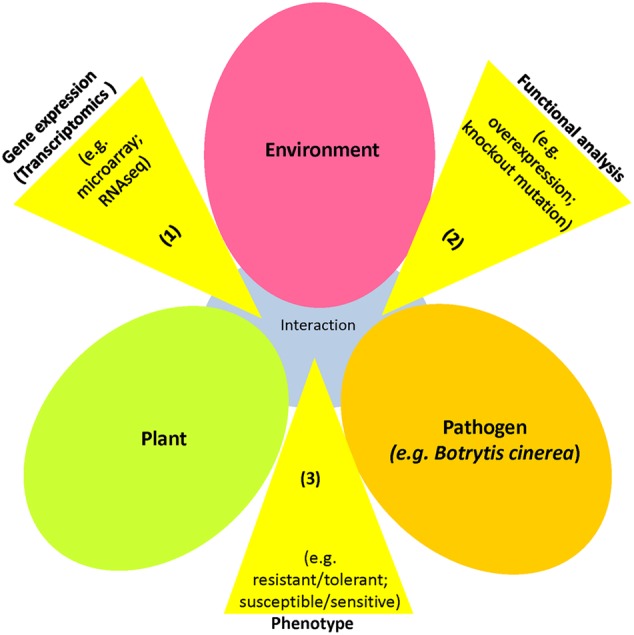
**An overview of reverse genetics approach used in gene discovery.** Disease triangle of plant-pathogen-environment interactions. In reverse genetics, the sequence of the gene is identified, but the function is not known. Steps of reverse genetic analysis: (1) Transcriptome analyses during the plant-pathogen interaction to identify differentially expressed genes; (2) Candidate genes can be identified and functionally characterized (e.g., overexpression, knockout); and (3) phenotypic effects of candidate genes can be determined (resistance/tolerance or susceptibility/sensitivity). Reverse genetics studies are commonly used to produce plant varieties resistant to pathogenic microbes, such as *Botrytis cinerea*.

Recently, transcriptomics studies and genetic mutagenesis have been developed to generate tagged mutants for reverse genetics purposes (**Figure 2**). Transcriptomics enables us to identify potential candidate genes functioning in plant defense, whereas mutant lines with knockout and/or overexpression traits allow us to elucidate their function in the defense response. *Botrytis-induced kinase 1* (*BIK1*), *responsive to dehydration 20* (*RD20*), *pentatricopeptide repeat protein for germination on NaCl* (*PGN*), and *expansin-like A2* (*EXLA2*) were identified as *B. cinerea*-responsive genes, and their mutants exhibited altered susceptibility to necrotrophic pathogens (Veronese et al., 2006; Laluk et al., 2011; AbuQamar et al., 2013; AbuQamar, 2014; Sham et al., 2015). Moreover, the *Mediator 18* (*MED18*), identified using RNAseq, was shown to modulate plant immunity and responses to hormones (Lai et al., 2014). These *B. cinerea*-responsive genes appear to be important in the pathogenesis of *B. cinerea*, as well as plant responses to various abiotic stressors (Lai et al., 2014). These findings confirm the existence of crosstalk in plant responses to *B. cinerea* infection and abiotic stress, involving various signaling hormone pathways, which affects photosynthesis, protein synthesis and transport, thereby highlighting the complexity of cellular signaling networks in plants (Birkenbihl et al., 2012; Windram et al., 2012; AbuQamar et al., 2016). The integration of genomics and transcriptomics, along with proteomics will identify biomarkers for biotic and abiotic stresses. This can be achieved by considering comparison of two (or more) different omic data sets (e.g., transcriptomic and proteomic data) to create a reference data set sharing the same functional context (Haider and Pal, 2013). This approach can build a dynamic model of functional features of biological processes/pathways involving transcripts and proteins.

## Proteomics

Protein identification can be done in a serial fashion to monitor the presence, absence or the overall quantity of a protein. Proteomic approaches can be used to investigate changes in protein levels under adverse stress conditions. Protein microarrays, for example, were successfully used to identify potential proteins interacting with calmodulin and calmodulin-like proteins (Popescu et al., 2007). However, a challenge in the construction of protein microarrays is that the proteins must be produced and purified from biological systems that allow proteins undergo posttranslational modifications and folding so that they retain their functions on the chip (Popescu et al., 2007). Proteins can also be separated using 1-dimensional (based on size only) or 2-dimensional (based on charge and size) protein gel electrophoresis (Gallagher, 2006; Rabilloud and Lelong, 2011), or chromatography (e.g., ultra-high speed MALDI-TOF and high mass resolution MALDI FTICR imaging MS) (Spraggins et al., 2016). A comparative proteomic analysis of two *B. cinerea* strains 1.11 and 2100 identified proteins that play crucial roles in their differential virulence, including housekeeping enzymes, such as malate and glyceraldehyde dehydrogenases (Fernández-Acero et al., 2007). In another research using shotgun proteomics, 126 proteins were altered in the proteome secreted by *B. cinerea*, of which 13 were pectinases that are involved in cell wall degradation (Shah et al., 2009). More recently, comparative proteomic analysis identified significant differences in the secretomes of *B. cinerea* strain B05.10 between pH 4–6 (Li et al., 2012). Proteins related to proteolysis were induced at pH 4, whereas cell wall degrading enzymes were accumulated at pH 6 (Li et al., 2012). Proteomics of tomato fruits infected by *B. cinerea* revealed changes in 186 proteins in mature green wild-type fruit, which were unaltered in red ripe (RR) wild-type and *ripening inhibited* (*rin*) mutant. However, fewer defense-related proteins were changed in mature green wild-type fruit than in RR and *rin* fruits (Shah et al., 2012).

## Metabolomics

Metabolomics may identify phenotypic effects of stresses on plants by measuring the abundance of metabolites, which fall downstream of genomic, transcriptomic and proteomic variations, and thus providing a dynamic measure of phenotypic responses to environmental cues (for reviews, see Hasanuzzaman et al., 2013; Hong et al., 2016). Metabolomic profiling is often performed with NMR, or MS, such as gas chromatography-MS (GC-MS) and liquid chromatography-MS (LC-MS) (for reviews, see Tuteja et al., 2011; Gathungu et al., 2014). Pathogen sensing, enzyme activities, and protein/antibodies and carbohydrate-binding screening can also be detected by carbohydrates and metabolites microarrays (Yadav et al., 2015). Metabolomic approaches have the ability to measure a broader array of small-molecules when plants are subjected to adverse conditions (Zhao et al., 2016). Primary metabolites, such as sugars, amino acids and Krebs cycle intermediates, are mainly involved in plant responses to abiotic stresses. Their changes are considered as indications of photosynthetic dysfunction and/or osmotic readjustment (Arbona et al., 2013). Secondary metabolites, on the other hand, respond to particular stress conditions, such as pathogens, antioxidants, ROS scavengers, coenzymes, and regulatory molecules.

The induction of secondary metabolites by several abiotic stressors could also be an effective mechanism of cross-protection against biotic threats, providing a link between abiotic and biotic stress responses. For example, *Arabidopsis* plants co-treated with UV-B and flagellin effector flg22 showed accumulation of flavonols and enhanced resistance to *B. cinerea* (Moura et al., 2010), indicating that induced flavonols might play a role in protecting plants against biotic stressors. Like other necrotrophic fungi, *B. cinerea* often produces unspecific phytotoxins, including secondary metabolites, as “killing” weapons to cells from a range of plant species. Over 40 clusters of genes were identified in *B. cinerea*, which were dedicated to the synthesis of polyketides, terpenes, non-ribosomal peptides and alkaloids, indicating that *B. cinerea* has the potential to produce many metabolites (Collado and Viaud, 2016). Global metabolites profiling using ^1^H NMR reveals significant metabolic variations between healthy and botrytized grape berries (Hong et al., 2012). Similar to healthy berries, botrytized ones accumulated high levels of proline, glutamate, arginine, and alanine; whereas unlike healthy ones, botrytized berries showed large degradation of phenylpropanoids, flavonoids, sucrose producing glycerol, gluconic acid, and succinate. Similarly, significant changes in primary and SM in tomato were reported to be associated with *B. cinerea* infection (Camañes et al., 2015), suggesting a prominent metabolic reprograming. The remarkable metabolic changes in *Arabidopsis*, grapes and tomato upon infection with *B. cinerea* cause metabolic perturbations both in the plant and the fungal pathogen.

## Conclusion

There is no doubt that omics is providing insights to the molecular mechanisms of plant resistance to pathogens and tolerance to environmental stresses for better disease management. Scientists are making a great effort to link genes with traits to improve resistance of cultivars and understand the mechanisms of disease resistance. Omics enables us to “translate” the complex interactions among genes (genomics), mRNA (transcriptomics), proteins (proteomics), and metabolites (metabolomics) into improvement of phenotypes, leading to enhanced crop productivity. Through omic technologies, the consistency and predictability of plant genetic engineering and breeding will be significantly improved by reducing the time and expense of producing resistant crops against *B. cnerea*.

## Author Contributions

SA and L-ST wrote the manuscript, with input and editing from KM.

## Footnotes

1 www.arabidopsis.org
